# A Review of Pyorrhea Alveolaris

**Published:** 1921-07

**Authors:** M. Rattner


### A REVIEW OF PYORRHEA ALVEOLARIS

BY M. RATTNER, D.D.S.

Looking through the literature, both medical and dental, on the subject of pyorrhea, one is surprised at the many unique ideas and explanations that different authors use to explain this disease.

A subject that is so much talked about and so little known is rather difficult to explain, and can not help but excite interest and create discussion.

Talking with several practitioners at the National Meeting in Boston, we were impressed by the fact that few dentists seem to know what pyorrhea is, and fewer want to know or care about recognizing it. A very limited number seem to care to spend the time necessary to find out a few of the cardinal principles involved in its recognition and treatment.

A business man had been told by his physician to go to a dentist and be treated for pyorrhea. When he told this to the dentist the latter remarked, "Oh! no, you haven't pyorrhea—you have Rigg's disease.". There may be some value in having so many different names for some diseases.

The early history of pyorrhea is not in any way clearly separated from that of the other dental diseases. Peridental diseases are referred to in the Bible and the Talmud and in Grecian and Roman literature until the first part of the eighteenth century, but there is no clearly drawn line between these and the other dental defects. The first distinct description of the clinical charactertistics that we notice was by the Frenchman, Pierre Fauchard, in 1728. From then on for almost a hundred and fifty years nothing practically was written about the subject. Magitot reopened the discussion about it when he referred to it as a constitutional disease.

This part of the nineteenth century was given to the discussions, considerations of this disease. Although all agreed upon the condition known as pyorrhea alveolaris, they did not at all agree as to the cause. The report of a commission of scientists of the Societe de Chirurgie of Paris did not settle matters any by their decision that it was due to constitutional defects.

For Germany, the most prominent man of this period was Robitzeck, of Vienna. In 1862 he devised a method for curing pyorrhea. His operation consisted in excising all the abnormally appearing gum tissue with a straight incision through the soft tissues down to the alveolar process. A scraping of the exposed alveolar bone followed the excission of the soft tissues and the patient was dismissed.

For America, the one who stands out pre-eminently in this branch of dentistry is John W. Riggs, of Hartford, Conn. In 1869 he published an article taking the position that pyorrhea alveolaris was caused by an ''irritation of calculi and other accretions upon teeth contiguous to or beneath the free margin of the gums.'' He was a confirmed localist.

After him we can enumerate quickly D. D. Smith, of Philadelphia, G. V. Black, of Chicago, and A. Witzel, of Germany, both localists. Eugene S. Talbot was among the first Americans who took into consideration both the local and constitutional forces that go together in causing this disease. He placed stress on infection and nutritional disturbances of the physiology and pathology of the underlying tissues, as well as calcular deposits, and on the factors involving evolution and environment.

The bacteriological part of pyorrhea alveolaris presents the most complex, the most varied and the most irritating part of the whole question. W. D. Miller, the father of scientific dentistry, was the first man to isolate the staphylococcus aureus and albus and the streptococcus pyogenes from pyorrheal pockets and described sixteen other types that he was not able to isolate. His conclusion was that pyorrhea may be produced by a wide group of organisms in varying combinations and is not in any case caused by a specific type of bacteria.

It was an Englishman that introduced the small-pox vaccine and it was left for another Englishman to introduce a pyorrhetic vaccine; Kenneth Goadby, of London, thought he could control this disease by using autogenous vaccines.

L. S. Medalia, of Boston, went a step further in his treatment of the disease and combined the vaccine treatment with oral prophylaxis, and in this way he succeeded in curing many cases of pyorrhea about ten years ago.

In 1916 the vaccine treatment was discredited when it was found that vaccine for pyorrhea was not a specific and its promiscuous use was dangerous.

Before the vaccine treatment had been completely discredited there arose a new method of treatment—a specific remedy for the scourge of pyorrhea was believed to be found in Emetin, founded on the amoeba theory. One need only to mention the names of the leading exponents—Drs. Barrett and Smith, of Philadelphia, and Drs. Bass and Johns, of New Orleans. Hartzell and others disproved these theories, and to prove that surgical prophylactic measure without the aid of Emetin would alone compete to cure pyorrhea by surgical procedures.

The last medicinal treatment was reported by Reed, Wright and White, of the United States Navy. They used succinamide of mercury, injecting it deeply into the gluteal muscles. Very little has been done for this treatment though, and therefore little can be said about its merits.

What is pyorrhea? So many have answered this question in diverse ways that it is difficult to give a real definition to a misnomer. One that would seem to take care of all the conditions is as follows:

It is a disease of the tissues surrounding the teeth; i. e., gums, gingivae, alveolar process, cementum and pericementum. It is characterized by infection and inflammation, and there may, or may not, be suppuration. It is a progressive resorption of the alveolar process and pericementum with a coincident recession of the gums and the formation of pockets in the later stage.

From its earliest recognition the profession was divided as to the causes of this malicious disease, and today we are nearly as much baffled by it as we were two hundred years ago. What has caused this difference of opinion? There is probably only one answer to this question, and that is, the unscientific manner in which the dental profession has up to recently studied the general pathological, physiological and histological studies belonging to our curriculum. This has lead to the unscientific hit and miss methods with which the dental and medical professions have gone about to solve this problem. Yet there are some simple measures that would help a correct diagnosis of the systemic, constitutional, and local causes, attributed to pyorrhea.

Every dental practitioner should make a small chart stating patient's name, age, occupation, hereditary inclinations and his general physical condition. Then, the condition of the mouth should be carefully notated. Anyone of the charactertistic diseases belonging to the etiology of pyorrhea—both local and constitutional—should be immediately called to the attention of the family physician.

On this chart there should be a report made of the conditions both systemic and local as they appear before and after treatment. The dental as well as the medical practitioners should then consult as to their findings and ideas.

The dentist should try and find out exactly at what period the patient first noticed abnormal conditions of his mouth. The physician should find out exactly when the systemic trouble was started, and each of them should record any improvement with treatment, and they should consult one another from time to time. Instead of this, we find that the dentist literally washes his hands from the patient when he sends him to the medical man! The doctor of medicine looks wisely into the patients oral cavity, notes the condition and immediately advises extraction.

In how many of our hospitals to-day will you find patients required or taught to take care of their mouths? It is a very hothouse of bacterial activity and is altogether disregarded and neglected notwithstanding the fact that focal infection is an established factor.

I can do no more than to set down the conditions both systemic and local that are found given in the different journals. It is rather peculiar to note that the medical man it not broadminded enough to look at the dental causes as to the general systemic causes—and the dental practitioner either has not had the advantages of recognizing the general conditions or has left them out altogether and looks only to the local and immediate causes.

Some of the general systemic conditions found coupled with pyorrhea are:

Tuberculosis—80% out of 1,776 cases.
Anemia—more than 50%.
Syphilis—no figures given.
Alimentary Toxemia—no figures given.
Diabetes—approximately 23%.
Gout—no figures given.
Nutritional disturbances—no figures given.
Faulty metabolism—no figures given.
Abnormal physical condition—no figures given.

No specific, systemic or organic disease involved.

The local causes really imply an interrupted blood supply, for by the circulatory apparatus the natural tone of the investing tissues is kept up. The tissues depend upon the cell and the latter depend upon the blood supply for their nourishment and existence, therefore, whenever there is an impediment to the natural circulation the cells lose their vitality and the tissues deteriorate.

The local causes are:

1. Trauma. Caused by—
    - (a) Ill-fitting crowns, inlays, fillings, bridges, etc.
    - (b) Malocclusion.
    - (c) Calculi—
        - (1) Salivary.
        - (2) Serumnal.
    - (d) Foods.

Trauma will cause an injury to the soft tissues; this in turn produces a hyperemia and stasis. Some necrosis follows of the gingivae with scission of the peridental fibres. The last stage is the deposition of food debris, calculi and bacterial activity.

2. Bacteria—
    - (a) Staphylococcus.
    - (b) Streptococcus.
    - (c) Diplococcus.
    - (d) Fusiform Bacilus.
    - (e) Spirochetae.
    - (f) Amoeba.

In every pyorrhetic pocket one will find bacteria of many varieties and forms. The cocci predominate, and the most important and specific for this disease are the staphylococus and streptococcus type. Some authors mention the fusiform bacilus, although Hartzel claims to have only heard of six cases due to this bacteria.

3. Oral Sepsis.

This will usually include an enormous amount of bacteria and food debris. The only picture mentally that one can derive from the term oral sepsis is a *dirty* or *filthy mouth.*

The recognition of this disease is by far the most essential. More pyorrhea goes by unnoticed than that which is recognized.

What is the cause of such a state of affairs? Generally the dentist is so indolent that he does not care to bring to the patients notice the looseness of the gums, the change in their color, and the slight deviations of attachments, because he fears he will not be able to cope with the situation successfully. It may be because there is more money and more glory in putting into the patient's mouth some piece of golden architecture.

There is no questioning the fact that early recognition insures a quick relief and a certain cure in most cases.

A few of the cardinal principles involved in the diagnosis of pyorrhea are given by Bunting, who lays stress upon the condition of the gums. His method of diagnosis is as follows:

1. *Color.* The normal gum tissue has a uniform color and any change in this uniformity indicates that the tissue is abnormal.

2. *Form.* The normal position of the gum tissue is about the necks of the teeth. The abnormal conditions are of two kinds:

(a) Retraction or shrinkage.

(b) Hypertrophy or swelling.

3. *Density.* Normally this tissue is firm, hard and resistant to touch. When injured or insulted they are soft, spungy and bleed easily.

4. *Line of attachment.* The normal tissue is firmly attached to the teeth. "True pyorrhea does not exist as long as the line of attachment of the soft tissues about the root remains normal. A space must exist between the root and the peridental tissues."

5. *Loosening of the teeth.* This statement is self-explanatory.

6. *Pus.* Such a case shows that there is an active process going on and the prognosis should be favorable.

7. *Radiographic findings.* In the hands of certain operators this has proven a very valuable diagnostic aid. This part is really up to the individual. I have seen several radiographs of one case that were interpreted differently. It is really a question of the angle at which the radiogram was taken and the individual method of interpretation.

8. *General physical condition.* This is a very important diagnostic feature, especially when one realizes the systemic diseases that occur with pyorrhea.

*The Treatment.* In judging literature we say that the test of good literature is the standard of usage. The same may be applied to pyorrhea. From the very time Riggs read his paper in 1869 up to the present there has only been one treatment that has survived—that of surgery! Many drugs have come to the fore in the last fifty years each one claiming to be a sure-cure remedy, only to be discarded in the end as a failure. So one by one they have all passed away. One can successfully claim to have treated pyorrhea only after the removal of all foreign matter about the teeth. That can be accomplished in one way—scaling. One must carefully and tenderly go over each tooth surface—go down as far as possible towards the apex and clean and smooth. The patient's aid must be enlisted and success can only be obtained when the dentist and patient work faithfully together.

There is one other surgical treatment that has been successfully tried, and that is the modified Robitzeck introduced by Zentler. His method is as follows:

"In excising the diseased gum tissue instead of a straight incision, by following the original festoons of the gums, going well into healthy tissue, leaving behind a scalloped edge. Sponge parts well to obtain clear view of the exposed surfaces of teeth and roots and these are again lightly curetted, making sure that all deposits and infectious accumulations are removed; the parts are swabbed with a 50% solution of the official tr. of iodin and a narrow strip (1/8 inch) of iodoform gauze is firmly packed over the entire surface operated upon, utilizing the interstices of teeth and roots to hold gauze in place. Remove gauze after 48 hours and according to the condition the packing may be repeated once or twice. After granulation forms in the parts, the mouth washed and tr. of iodin are discontinued by the patient, the exposed surfaces of the teeth and roots can be given the prophylactic treatment and patient instructed in self-care of the mouth."

Whether one treats pyorrhea alveolaris by Zentler's method or by the ordinary scaling and polishing, we must keep in mind the old axium that that method is best which gives the best results in one's own hands.

The surgical treatments are more painful and more responsive; therapeutic treatments give slower results and are usually less painful. Faithful prophylaxis is essential in conjunction with both surgical and medical treatments. Both surgical and medical treatments can be applied successfully by both treatments, but thoroughness with excessive applications may do much harm.

Bibliography:

Pyorrhea Alveolaris, R. W. Bunting.
Dental Items of Interest, Zentler, March, 1919.
N. Y. Medical Journal, Benedict, June, 1918.
N. Y. Medical Journal, Merritt, March, 1917.
Dentistry in Bible and Talmud, S. Grief.
N. Y. Medical Journal, September, 1916.
Journal American Med. Assn., Feb. 10, 1917.
Journal American Med. Assn., Nov. 9, 1918.

